# Effectiveness of a locally produced ready‐to‐use supplementary food in preventing growth faltering for children under 2 years in Cambodia: a cluster randomised controlled trial

**DOI:** 10.1111/mcn.12896

**Published:** 2019-12-29

**Authors:** Bindi Borg, Daream Sok, Seema Mihrshahi, Mark Griffin, Chhoun Chamnan, Jacques Berger, Arnaud Laillou, Nanna Roos, Frank T. Wieringa

**Affiliations:** ^1^ School of Public Health, Faculty of Medicine University of Sydney Sydney Australia; ^2^ Department of Nutrition, Exercise and Sports, Faculty of Science University of Copenhagen Copenhagen Denmark; ^3^ School of Public Health University of Queensland Brisbane Australia; ^4^ Department of Fisheries Post‐Harvest Technologies and Quality Control, Fisheries Administration Ministry of Agriculture, Forestry and Fisheries Phnom Penh Cambodia; ^5^ UMR‐204 Nutripass Institut de Recherche pour le Développement, IRD/UM/SupAgro Montpellier France; ^6^ Child Survival and Development Section UNICEF Phnom Penh Cambodia

**Keywords:** childhood malnutrition, fish, growth faltering, lipid‐based nutrient supplement (LNS), ready‐to‐use supplementary food (RUSF)

## Abstract

This cluster randomised controlled trial tested the effectiveness of a locally produced, fish‐based, ready‐to‐use supplementary food (RUSF) to prevent growth faltering (decline in z‐scores). Cambodian infants (n= 485), aged 6 to 11 months, were randomised by site to receive the RUSF, Corn‐Soy Blend++ (CSB++), micronutrient powders (MNP), or no supplement (control). The intervention was for 6 months. In unadjusted analysis, the control group had statistically significantly decreased weight‐for‐age z‐scores (WAZ; ‐0.02, 95%CI = ‐0.03 ‐ ‐0.01, *P*= 0.001) and height‐for‐age z‐scores (HAZ; ‐0.07, 95%CI = ‐0.09 ‐ ‐0.05, *P* < 0.001), and increased mid‐upper arm‐circumference (MUAC; 0.02cm, 95%CI = 0.01 ‐ 0.04, *P* = 0.010), but no statistically significant change in weight‐for‐height z‐scores (WHZ). The RUSF group did not differ significantly from the control for WAZ, HAZ or WHZ (in other words, WAZ and HAZ decreased and WHZ did not change), but had increased MUAC in comparison to the control (0.04cm, 95%CI = 0.01 ‐ 0.06, *P* = 0.008). There were no statistically significant differences between the RUSF group and the CSB++ or MNP groups with respect to WAZ, HAZ, WHZ or MUAC. Interestingly, in adjusted analysis, low consumers of RUSF had increased WAZ, WHZ and MUAC (0.03, 95%CI = 0.01‐0.06, *P* = 0.006; 0.04, 95%CI = 0.01‐0.08, *P* = 0.026; and 0.05cm, 95%CI = 0.02‐0.09, *P* = 0.004, respectively) compared with the control. The novel RUSF, particularly in small quantities, protected against ponderal growth faltering, but the improvements were of limited clinical significance.

## 1 BACKGROUND AND RATIONALE

Undernutrition contributes to almost half of all deaths in children under 5 years (Black et al., 2013). In Cambodia, despite impressive economic growth, high rates of undernutrition persist (NIS et al., 2015). In the 2014 Cambodian Demographic and Health Survey (DHS), almost one‐third (32%) of children under 5 years were stunted, 10% were wasted and 24% were underweight (NIS et al., 2015). The majority of growth faltering, indicated by a decline in z‐scores (Victora, de Onis, Hallal, Blössner, & Shrimpton, 2010) in Cambodia occurs from 6 to 20 months (Dewey & Huffman, 2009; NIS et al., 2015). Poor complementary feeding practices are often implicated in the growth faltering observed in low‐ and middle‐income countries (Ferguson et al., 2018). *Borbor* (white rice porridge*,* the traditional weaning food in Cambodia) has inadequate energy and micronutrient nutrient density to sustain adequate growth velocity in the first 2 years of life (Black et al., 2008; Ferguson et al., 2018). Nutrition‐specific interventions aimed at improving complementary feeding seem warranted (Black et al., 2013; Pham et al., 2012). High energy, nutrient dense specialised foods can be used to prevent growth faltering and promote improved linear growth and weight gain among children (Bhutta et al., 2013; Pee & Bloem, 2009; Golden, 2009).

The development of affordable, acceptable and effective specialised foods, and their comparison with existing products in terms of their potential for preventing growth faltering responds to a need noted by researchers (de Pee & Bloem, 2009; Lazzerini, Rubert, & Pani, 2013). In Cambodia prior to 2013, various supplementary or therapeutic foods had been used or trialled. Corn‐Soy Blend Plus Plus (CSB++, also called SuperCereal Plus, the standard supplementary food that WFP provides to children aged 6 months to 2 years to prevent undernutrition), BP‐100™ and Plumpy'Nut™ had limited acceptability or effectiveness (Boudier, 2009; WFP, 2014; Wieringa, 2014). Micronutrient powders (MNP), while acceptable and effective at improving micronutrient status, did not have any impact on growth (Jack et al., 2012). Therefore, in mid‐2013, UNICEF engaged the French National Research Institute for Sustainable Development (IRD), and the Cambodian Department of Fisheries Post‐harvest Technologies and Quality (DFPTQ), to develop a locally produced ready‐to‐use supplementary food (RUSF). The aim was to develop an RUSF that would be more acceptable, effective and affordable than previously tested or used products (Sigh et al., 2018).

Many specialised foods, including CSB++, use milk or whey powder as the animal‐source food (Adu‐Afarwuah, Lartey, Zeilani, & Dewey, 2011; Nga et al., 2013), but in Cambodia, milk is an expensive, imported ingredient. Thus, it was decided to replace milk with fish, which is inexpensive, readily available and highly acceptable in Cambodia (Vilain, Baran, Gallego, & Samadee, 2016). It had previously been demonstrated that fish protein supported linear growth to the same extent as milk protein in a locally produced complementary food in Cambodia (Skau et al., 2015). Since lipid‐based nutrient supplements (LNSs) are particularly promising (de Pee & Bloem, 2009; de Pee, Manary, & Ashorn, 2011), the novel ready‐to‐use supplementary food (RUSF) was formulated as an LNS snack. In June 2015, the RUSF was tested for acceptability in comparison to CSB++ and MNP. The acceptability trial demonstrated that children would eat the RUSF and that caregivers ranked it highly (Borg et al., 2019). Here, we report on the effectiveness of the RUSF in preventing growth faltering for children aged 6 to 17 months, in comparison to CSB++, MNP, and an unsupplemented control group. The main outcomes of interest are weight‐for‐age z‐score (WAZ), height ‐for‐age z‐score (HAZ), weight‐for‐height z‐score (WHZ), and mid‐upper arm circumference (MUAC).

## 2 METHODS

### 2.1 Study design and setting

The design and methods are detailed in the published protocol (Borg et al., 2017) and briefly described here. The trial took place from February to October 2016. It was a prospective, non‐blinded, cluster randomised controlled trial among infants that were 6 to 11 months of age at inclusion. It aimed to establish the superiority of the novel RUSF, using CSB++, and MNP as active comparators and the standard diet as a control. The trial was conducted in peri‐urban Phnom Penh (Mekong Operational District), which has a large population of urban poor. Peri‐urban children under 5 years experienced higher rates of underweight (36%) and stunting (29%) than the 25% and 19% reported for Phnom Penh, respectively (UNICEF/ PIN, 2014; NIS et al., 2011). Twenty‐eight sites were allocated to one of the RUSF, CSB++, MNP, or control groups.

### 2.2 RUSF formulation

The RUSF was based on the recommended nutritional guidelines for ready‐to‐use therapeutic foods (Dewey, 2009; FAO/WHO, 2016). It was produced locally, using local ingredients including small freshwater fish, soy, mung beans and coconut. The RUSF paste was piped into hollow, cylindrical wafers which are a popular Cambodian snack. All processing was conducted in certified facilities, and microbiological safety testing was conducted regularly. The ingredients of the RUSF and the comparators are detailed in Tables A1 and A2, and in the acceptability and effectiveness protocols (Borg et al., 2017; Borg et al., 2018). The RUSF was provided as a medium quantity supplementary food, that is, providing 50‐100% of the child's daily energy requirements (i.e. 250 to 500 kcal) excluding breastfeeding (Gera, Pena‐Rosas, Boy‐Mena, & Sachdev, 2017). This was 40‐110g of RUSF per day, depending on the child's age. The nutrient profiles of all the supplements were similar in terms of multiple micronutrients. The RUSF and CSB++ were similar in terms of energy, protein, carbohydrate, and lipid content.

### 2.3 Outcomes and their measurement

The main outcomes of interest were anthropometric measures calculated using World Health Organisation (WHO) 2006 standards (ANTHRO version 3.2.2 January 2011) and expressed as z‐scores, namely WAZ, HAZ, and WHZ, along with MUAC in centimetres (cm). Data was collected by a dedicated anthropometrist, supported by a dedicated anthropometric data collector, both of whom received initial and follow up training.

### 2.4 Randomisation and allocation concealment

Participants were not individually randomised. Randomisation of the interventions occurred at site level to ensure better compliance by avoiding potentially confounding social interaction, such as inter‐household sharing of different foods (Van Hoan, Van Phu, Salvignol, Berger, & Trèche, 2009). Using UNICEF data on health centre coverage, potential sites and their populations were listed. Sites were then randomly allocated to one of the foods, using an Excel random number table and a randomised incomplete block design. The principal researcher generated the allocation sequence. Seven sites were allocated to each arm, for a total of 28 sites. One site yielded only one participant, who dropped out, leaving 27 sites at the end of the study.

### 2.5 Sample size

Based on the assumptions of a difference in mean z‐scores of 0.1 between the groups (95%CI), a standard deviation (SD) of 0.8, and of children providing five measurements (out of a possible total of seven), with a precision of 0.05 and power of 0.8, an overall required sample size of 424 children, or 106 children per group, was calculated. We assumed an attrition of 25%, for a total sample of 530 or 133 children per group. This sample size was comparable to similar effectiveness studies (Jack et al., 2012; Kuusipalo, Maleta, Briend, Manary, & Ashorn, 2006; Lin, Manary, Maleta, Briend, & Ashorn, 2008; Nga et al., 2013; Pham et al., 2012).

### 2.6 Eligibility criteria, recruitment, enrolment and consent

Healthy singletons aged 6 to 11 months were enrolled. Village health volunteers invited potential caregivers and children to participate. The data collection team used a screening form to assess initial eligibility (e.g. based on age, singleton status, and willingness to participate). Most caregivers had a birth certificate or immunisation card with the child's date of birth, or if not, they were asked if they knew the child's birthdate or age. Children who were ill, severely acutely malnourished (WHZ <‐3 and/or MUAC<11.5cm), obese (WHZ >3), severely anaemic (Hb<70g/l), or had known food intolerances, were excluded and referred for treatment as necessary. Caregivers of eligible participants signed or fingerprinted consent for their children to participate.

### 2.7 Data collection

Baseline data including demographics; morbidity; anthropometric measures; biochemical samples (blood, stool); dietary data; and developmental milestone achievement was collected. Baseline and monthly follow up data were collected at community sites (e.g. health volunteers' homes, pagodas) or health centres by a team of trained data collectors. Participants in the intervention groups were provided with a 1‐month supply of the food or supplement to which their site had been allocated. Thereafter, data collection and food distribution were conducted monthly for 6 months. Anthropometric measurements included weight to the nearest 0.1 kg (SECA scale), recumbent length to the nearest 0.1 cm (wooden UNICEF height board), and mid‐upper arm circumference (MUAC) to the nearest 1mm (flexible UNICEF insertion tape).

Caregivers were given incentives to participate, including cost of transport, and/or a small gift such as a towel or baby item. Health promotion messaging was not an explicit part of the project. Every month, at the end of data collection, all caregivers were reminded to continue if breastfeeding; to feed their baby normally, three to five times daily; and to maintain adequate hygiene (safe stool disposal, handwashing after defaecation and before eating/feeding). Caregivers in the intervention arms were reminded to feed their baby the supplement or supplementary food in the recommended dosage. Caregivers in the RUSF and CSB++ groups were reminded that the supplementary foods were an extra snack in addition to regular feeding.

### 2.8 Statistical analysis

Data was analysed in STATA version 13.1. Comparisons between food types for children enrolled at baseline (n = 485) were made using one‐way ANOVA for continuous variables (reported as mean and SD), Kruskal‐Wallis rank test for non‐normally distributed continuous variables (reported as median and interquartile range, IQR), and chi‐squared for categorical variables, (reported as n and %). These results are reported in Table 1.

**Table 1:** Baseline Characteristics of Enrolled Children and Their Caregivers

Characteristics at baseline	Total (N=485^a^)	Control (n=127, 26%)	RUSF (n=128, 26%)	CSB++ (n=123, 25%)	MNP (n=107, 22%)	*P*‐value^b^
Age in months at baseline, mean (SD)	8.5 (1.7)	8.4 (1.7)	8.4 (1.8)	8.6 (1.8)	8.5 (1.7)	0.754
Female, n (%)	233 (48.0%)	73 (57.5%)	53 (41.4%)	50 (40.7%)	57 (53.3%)	0.014*
Weight in kg, mean (SD)	7.71 (1.05)	7.76 (1.09)	7.69 (0.95)	7.69 (1.07)	7.67 (1.09)	0.912
Length in cm, mean (SD)	68.7 (3.8)	68.8 (3.9)	69.1 (3.9)	68.6 (3.9)	68.3 (3.4)	0.478
Weight‐for‐age Z‐score (WAZ), mean (SD)	‐0. 80 (1.06)	‐0.66 (1.09)	‐0.83 (0.97)	‐0.92 (1.05)	‐0.81 (1.14)	0.283
Underweight (<‐2), n (%)	62 (12.8%)	17 (13.4%)	15 (11.7%)	17 (13.8%)	13 (12.2%)	0.955
Height‐for‐age Z‐score (HAZ), mean (SD)^c^	‐0.70 (1.17)	‐0.53 (1.18)	‐0.58 (1.15)	‐0.89 (1.19)	‐0.81 (1.11)	0.040*
Stunted (<‐2), n (%)	61 (12.6%)	15 (11.8%)	12 (9.4%)	18 (14.6%)	16 (15.0%)	0.516
Weight‐for‐height Z‐score (WHZ), mean (SD)	‐0.48 (1.01)	‐0.40 (1.05)	‐0.60 (0.98)	‐0.48 (0.93)	‐0.42 (1.09)	0.399
Wasted, moderately acutely malnourished (<‐2), n (%)	23 (4.7%)	4 (3.2%)	9 (7.0%)	6 (4.9%)	4 (3.8%)	0.486
Mid‐upper arm circumference (MUAC) in cm, mean (SD)	14.2 (1.1)	14.2 (1.1)	14.2 (1.0)	14.2 (1.0)	14.3 (1.1)	0.860
Low MUAC (<12.5cm), n (%)	20 (4.1%)	6 (4.7%)	4 (3.1%)	5 (4.1%)	5 (4.7%)	0.915
Birthweight, kg, mean (SD)	3.00 (0.47)	2.96 (0.50)	3.04 (0.46)	3.00 (0.47)	2.99 (0.46)	0.608
Low birthweight (<2.5kg), n (%)	62 (13.1%)	19 (15.3%)	13 (10.2%)	16 (13.7%)	14 (13.3%)	0.670
Iron status						
Iron deficient at baseline (ferritin ≤15μg/L), n (%)	85 (20.1%)	17 (15.2%)	29 (25.9%)	20 (19.1%)	19 (20.0%)	0.250
Iron replete at baseline (ferritin ≥ 50μg/L), n (%)	104 (24.6%)	35 (31.3%)	20 (17.9)	27 (25.7%)	22 (23.2%)	0.133
Diarrhoea in past 2 weeks, n (%)	154 (31.8%)	42 (33.1%)	43 (33.6%)	32 (26.0%)	37 (34.6%)	0.464
Prelacteal feeding, n (%)	236 (49.1%)	61 (48.0%)	55 (43.3%)	63 (51.6%)	57 (54.3%)	0.237
Exclusive breastfeeding < 3 months, n (%)	276 (57.4%)	76 (60.3%)	60 (47.2%)	73 (59.4%)	67 (63.8%)	0.107
Started complementary feeding <3 months, n (%)	51 (10.6%)	13 (10.5%)	11 (8.6%)	13 (10.6%)	14 (13.2%)	0.728
Still breastfeeding at baseline, n (%)	306 (63.5%)	81 (63.8%)	84 (66.7%)	79 (64.2%)	62 (58.5%)	0.631
Drank from bottle with nipple in past 24hrs, n (%)	401 (83.7%)	101 (80.8%)	110 (85.9%)	105 (85.4%)	85 (82.5%)	0.660
Minimum dietary diversity in past 24hrs, n (%)	144 (29.7%)	35 (27.6%)	46 (35.9%)	36 (29.3%)	27 (25.2%)	0.296
Mother's BMI, mean (SD)	22.5 (4.2)	21.9 (3.6)	23.0 (4.3)	22.3 (4.4)	23.1 (4.2)	0.099
Underweight at baseline (BMI <18.5), n (%)	72 (17.9%)	21 (18.8%)	16 (15.4%)	25 (25.0%)	10 (11.6%)	0.101
Caregiver:						
Never attended school, n (%)	56 (11.6%)	10 (7.9%)	10 (7.8%)	17 (13.8%)	19 (17.8%)	0.106
Attended primary school, n (%)	231 (47.6%)	62 (48.8%)	68 (53.1%)	51 (41.5%)	50 (46.7%)	0.106
Attended high school or higher, n (%)	198 (40.8%)	55 (43.3%)	50 (39.1%)	55 (44.7%)	38 (35.5%)	0.106
Shared toilet, n (%)	81 (17.2%)	26 (20.5%)	22 (17.9%)	21 (18.0%)	12 (11.7%)	0.353
Unsafe stool disposal, n (%)	351 (72.7%)	97 (76.4%)	92 (71.9%)	74 (60.7%)	88 (83.0%)	0.001**
Poor card holder, n (%)	77 (15.9%)	12 (9.5%)	23 (18.1%)	25 (20.3%)	17 (15.9%)	0.102
Household income, $, median (IQR)^d^	225 (150‐300)	245 (200‐375)	200 (150‐300)	200 (150‐300)	225 (150‐300)	0.141

Asterisks highlight significant *P*‐values:

* <0.05,

** < 0.01,

*** <0.001.

a Not all children provided complete information for each variable.

b P‐values were computed by comparison of different food types. For continuous variables (reported as mean and SD), comparison between food types was made using one‐way ANOVA. For categorical variables, (reported as n and %) comparison was made using chi‐squared.

c Histogram of all four arms of HAZ at baseline shows sufficient overlap. Therefore, the mixed effect model will be able to account for baseline difference.

d Non‐normally distributed, therefore quoted median (IQR), *P*‐value calculated using Kruskal‐Wallis rank test.

Comparisons between baseline and endline anthropometric measures for the different food types for children with baseline and endline data (n = 292) were made using one‐way ANOVA for continuous variables, and Kruskal‐Wallis rank test for continuous variables for which homogeneity of variance was not observed. Changes in proportion from baseline to endline (%) were calculated by subtracting baseline proportion from endline proportion, and *P*‐values were calculated using Pearson's chi‐squared test. These results are reported in Table 2.

**Table 2:** Change in Anthropometric Measures from Baseline to Endline for Children with Baseline and Endline Measurements

Change in anthropometric measures, baseline to endline	Total (N=292)	Control (n=77, 26%)	RUSF (n=76, 26%)	CSB++ (n=59, 20%)	MNP (n=80, 27%)	*P*‐value
Height in cm, mean (SD)						
At baseline, mean (SD)	68.5 (4.0)	68.9 (4.0)	69.1 (4.0)	67.9 (4.3)	68.1 (3.6)	0.198
At endline, mean (SD)	75.0 (3.8)	75.4 (4.0)	75.5 (3.7)	74.3 (4.2)	74.6 (3.2)	0.208
Difference baseline to endline, mean (SD)	6.5 (1.6)	6.7 (1.7)	6.6 (1.4)	6.4 (1.8)	6.5 (1.6)	0.717^a^
Weight in kg, mean (SD)						
At baseline, mean (SD)	7.64 (1.02)	7.74 (1.04)	7.67 (0.91)	7.54 (1.07)	7.59 (1.07)	0.669
At endline, mean (SD)	8.89 (1.16)	9.00 (1.28)	9.00 (1.13)	8.81 (1.16)	8.80 (1.05)	0.642
Difference baseline to endline, mean (SD)	1.25 (0.59)	1.23 (0.62)	1.30 (0.58)	1.26 (0.64)	1.20 (0.52)	0.779
Weight‐for‐age Z‐score (WAZ), mean (SD)						
At baseline, mean (SD)	‐0.84 (1.02)	‐0.67 (1.03)	‐0.85 (0.93)	‐1.03 (0.98)	‐0.88 (1.11)	0.245
At endline, mean (SD)	‐0.93 (1.04)	‐0.81 (1.09)	‐0.87 (1.05)	‐1.08 (1.01)	‐1.00 (0.99)	0.432
Difference baseline to endline, mean (SD)	‐0.08 (0.58)	‐0.14 (0.57)	‐0.01 (0.55)	‐0.05 (0.66)	‐0.11 (0.56)	0.541
Underweight (WAZ < ‐2), n (%)						
At baseline, n (%)	36 (12.3%)	10 (13.0%)	8 (10.5%)	7 (11.9%)	11 (13.8%)	0.936
At endline, n (%)	39 (13.4%)	8 (10.4%)	8 (10.5%)	9 (15.3%)	14 (17.5%)	0.479
Difference baseline to endline^b^, %	1.0%	‐2.6%	0.0%	3.4%	3.8%	0.870
Height‐for‐age Z‐score (HAZ), mean (SD)						
At baseline, mean (SD)	‐0.74 (1.19)	‐0.48 (1.21)	‐0.60 (1.13)	1.09 (1.20)	‐0.87 (1.15)	0.012
At endline, mean (SD)	‐1.04 (1.20)	‐0.85 (1.28)	‐0.84 (1.17)	‐1.38 (1.26)	‐1.16 (1.06)	0.021
Difference baseline to endline, mean (SD)	‐0.30 (0.76)	‐0.37 (0.93)	‐0.24 (0.70)	‐0.29 (0.81)	‐0.29 (0.58)	0.896^a^
Stunted (HAZ < ‐2), n (%)						
At baseline, n (%)	41 (14.0%)	8 (10.4%)	8 (10.5%)	11 (18.6%)	14 (17.5%)	0.326
At endline, n (%)	66 (22.6%)	17 (22.1%)	12 (15.8%)	18 (30.5%)	19 (23.8%)	0.241
Difference baseline to endline^b^, %	8.6%	11.7%	5.3%	11.9%	6.3%	0.877
Weight‐for‐height Z‐score (WHZ), mean (SD)						
At baseline, mean (SD)	‐0.50 (0.99)	‐0.45 (1.03)	‐0.62 (0.93)	‐0.45 (0.93)	‐0.46 (1.09)	0.657
At endline, mean (SD)	‐0.59 (1.02)	‐0.55 (1.02)	‐0.65 (1.07)	‐0.55 (0.96)	‐0.61 (1.03)	0.914
Difference baseline to endline, mean (SD)	‐0.10 (0.73)	‐0.10 (0.79)	‐0.03 (0.68)	‐0.10 (0.79)	‐0.16 (0.68)	0.770
Wasted (WHZ < ‐2), n (%)						
At baseline, n (%)	13 (4.5%)	4 (5.2%)	4 (5.3%)	2 (3.4%)	3 (3.8%)	0.926
At endline, n (%)	25 (8.6%)	7 (9.1%)	5 (6.6%)	4 (6.8%)	9 (11.3%)	0.709
Difference baseline to endline^b^, %	4.1%	3.9%	1.3%	3.4%	7.5%	0.826
Mid‐upper arm circumference (MUAC) in cm, mean (SD)						
At baseline, n (%)	14.2 (1.0)	14.3 (1.0)	14.1 (1.0)	14.1 (1.0)	14.2 (1.1)	0.678
At endline, n (%)	14.4 (1.1)	14.4 (1.1)	14.3 (1.2)	14.4 (1.1)	14.3 (1.1)	0.990
Difference baseline to endline, %	0.2 (0.8)	0.1 (0.8)	0.2 (0.9)	0.3 (0.8)	0.1 (0.9)	0.467
Low MUAC, (<12.5cm), n (%)						
At baseline, n (%)	10 (3.4%)	1 (1.3%)	4 (5.3%)	2 (3.4%)	3 (3.8%)	0.603
At endline, n (%)	10 (3.4%)	1 (1.3%)	3 (4.0%)	2 (3.4%)	4 (5.0%)	0.633
Difference baseline to endline^b^, %	0.0%	0.0%	‐1.3%	0.0%	1.3%	0.963

*P*‐values were computed by comparison of different food types. For continuous variables (reported as mean and SD), comparison between food types was made using one‐way ANOVA.

a In the case of HAZ and change in mean height from baseline to endline, homogeneity of variance was not observed, therefore Kruskal‐Wallis method was used. For categorical variables, (reported as n and %, or %) comparison was made using chi‐squared.

b Changes in proportion from baseline to endline (%) were calculated by subtracting baseline proportion from endline proportion, and *P*‐values were calculated using Pearson's chi‐squared test. Errors are due to rounding.

A mixed effects linear regression model was fit for each anthropometric outcome for children who had baseline and endline data to determine whether there were statistically significant differences in the changes in anthropometric outcomes from baseline to endline for the interventions compared to each of the other groups. The model adjusted for clustering by person and site, and used month as an interaction term to account for monthly follow‐up measures. Baseline values were accounted for in the model within each food group. The model included parameters for the slope of the line in the control group with respect to time, and the change in the slope between the intervention versus the control group. Children who ate less than 75% of the monthly food supplied were considered to have low consumption, while 75% or more was high consumption. These results are reported in Table 3.

**Table 3:** Change in anthropometric outcomes from baseline to endline comparing each pair of groups, for children with baseline and endline data

Change in anthropometric outcomes from baseline to endline	WAZ Coefficient (95% CI, *P*‐value)	HAZ Coefficient (95% CI, *P*‐value)	WHZ Coefficient (95% CI, *P*‐value)	MUAC (cm) Coefficient (95% CI, *P*‐value)
UNADJUSTED (n=292)				
Month^a^	‐0.02 (‐0.03 ‐ ‐0.01, 0.001**)	‐0.07 (‐0.09 ‐ ‐0.05, < 0.001***)	‐0.01 (‐0.03‐0.01, 0.231)	0.02 (0.01‐0.04, 0.010*)
RUSF x month^b^ versus control	0.02 (<‐0.01‐0.03, 0.083)	0.01 (‐0.01‐0.04, 0.312)	0.01 (‐0.01‐0.04, 0.373)	0.04 (0.01‐0.06, 0.008**)
RUSF x month^b^ versus CSB++	< 0.01 (‐0.02‐0.02, 0.858)	0.02 (‐0.01‐0.05, 0.224)	< ‐0.01 (‐0.03‐0.03, 0.897)	< 0.01 (‐0.02‐0.03, 0.793)
RUSF x month^b^ versus MNP	0.01 (‐0.01‐0.03, 0.244)	<0.01 (‐0.03‐0.03, 0.988)	0.02 (‐0.01‐0.05, 0.159)	0.03 (0.01‐0.06, 0.018)
CSB++ x month^b^ versus control	0.01 (‐0.01‐0.03, 0.151)	< ‐0.01 (‐0.04‐0.03, 0.778)	0.01 (‐0.01‐0.04, 0.337)	0.03 (<0.01‐0.06, 0.027*)
CSB++ x month^b^ versus MNP	0.01 (‐0.01‐0.03, 0.366)	‐0.02 (‐0.05‐0.01, 0.225)	0.02 (‐0.01‐0.05, 0.150)	0.03 (<‐0.01‐0.06, 0.053)
MNP x month^b^ versus control	0.01 (‐0.01‐0.02, 0.562)	0.01 (‐0.01‐0.04, 0.315)	‐0.01 (‐0.03‐0.02, 0.605)	<0.01 (‐0.02‐0.03, 0.756)
ADJUSTED (n=235^c^)				
Month^a^	‐0.03 (‐0.04 ‐ ‐0.01, < 0.001***)	‐0.06 (‐0.08 ‐ ‐0.04, < 0.001***)	‐0.03 (‐0.05 ‐ <‐0.01, 0.017*)	0.02 (<‐0.01‐0.03, 0.109)
RUSF high consumers x month^b^				
Versus control	0.03 (‐0.01‐0.06, 0.140)	< ‐0.01 (‐0.07‐0.06, 0.917)	0.03 (‐0.02‐0.09, 0.263)	0.08 (0.03‐0.13, 0.003**)
Versus CSB++	‐0.01 (‐0.05‐0.03, 0.714)	0.04 (‐0.03‐0.11, 0.238)	‐0.04 (‐0.10‐0.02, 0.182)	0.01 (‐0.04‐0.07, 0.703)
Versus MNP	0.01 (‐0.03‐0.05, 0.590)	‐0.02 (‐0.09‐0.05, 0.554)	0.02 (‐0.04‐0.08, 0.423)	0.03 (‐0.02‐0.08, 0.279)
RUSF low consumers x month^b^				
Versus control	0.03 (0.01‐0.06, 0.006**)	0.01 (‐0.03‐0.06, 0.596)	0.04 (0.01‐0.08, 0.026*)	0.05 (0.02‐0.09, 0.004**)
Versus CSB++	< ‐0.01 (‐0.03‐0.03, 0.990)	0.06 (0.01‐0.11, 0.031*)	‐0.03 (‐0.08‐0.02, 0.195)	‐0.02 (‐0.06‐0.03, 0.469)
Versus MNP	0.02 (‐0.01‐0.04, 0.215)	‐0.01 (‐0.05‐0.04, 0.834)	0.04 (‐0.01‐0.08, 0.103)	<0.01 (‐0.04‐0.04, 0.863)
CSB++ high consumers x month^b^				
Versus control	0.07 (0.03‐0.10, < 0.001***)	‐0.03 (‐0.09‐0.03, 0.337)	0.11 (0.05‐0.16, < 0.001***)	0.09 (0.04‐0.14, 0.001**)
Versus RUSF	0.04 (<‐0.01‐0.07, 0.053)	‐0.04 (‐0.10‐0.03, 0.259)	0.07 (0.01‐0.12, 0.027*)	0.03 (‐0.03‐0.08, 0.312)
Versus MNP	0.05 (0.01‐0.09, 0.006**)	‐0.05 (‐0.11‐0.02, 0.158)	0.10 (0.04‐0.16, 0.001**)	0.04 (‐0.01‐0.09, 0.154)
CSB++ low consumers x month^b^				
Versus control	0.01 (‐0.02‐0.04, 0.515)	‐0.06 (‐0.11 ‐ <‐0.01, 0.037*)	0.05 (<0.01‐0.10, 0.031*)	0.05 (0.01‐0.10, 0.014*)
Versus RUSF	‐0.02 (‐0.05‐0.01, 0.181)	‐0.06 (‐0.12 ‐ ‐0.01, 0.029*)	0.01 (‐0.04‐0.06, 0.659)	‐0.01 (‐0.05‐0.04, 0.792)
Versus MNP	‐0.01 (‐0.04‐0.03, 0.686)	‐0.07 (‐0.13 ‐ ‐0.02, 0.012*)	0.04 (‐0.01‐0.09, 0.091)	0.00 (‐0.04‐0.05, 0.833)
MNP high consumers x month^b^				
Versus control	0.04 (0.01‐0.06, 0.005**)	0.03 (‐0.02‐0.08, 0.192)	0.03 (‐0.02‐0.07, 0.208)	0.06 (0.02‐0.09, 0.004**)
Versus RUSF	0.01 (‐0.02‐0.03, 0.688)	0.02 (‐0.03‐0.08, 0.361)	‐0.01 (‐0.06‐0.03, 0.572)	0.00 (‐0.05‐0.04, 0.870)
Versus CSB++	< 0.01 (‐‐.03‐0.03, 0.811)	0.08 (0.02‐0.13, 0.006*)	‐0.05 (‐0.10 ‐ <0.01, 0.060)	‐0.01 (‐0.06‐0.03, 0.625)
MNP low consumers x month^b^				
Versus control	‐0.01 (‐0.04‐0.02, 0.433)	< ‐0.01 (‐0.06‐0.05, 0.923)	‐0.02 (‐0.07‐0.03, 0.460)	0.04 (‐0.01‐0.08, 0.087)
Versus RUSF	‐0.04 (‐0.07 ‐ ‐0.01, 0.008**)	‐0.01 (‐0.07‐0.05, 0.736)	‐0.06 (‐0.11 ‐ ‐0.01, 0.026*)	‐0.02 (‐0.07‐0.02, 0.364)
Versus CSB++	‐0.04 (‐0.08 ‐ ‐0.01, 0.009**)	0.04 (‐0.02‐0.10, 0.168)	‐0.09 (‐0.15 ‐ ‐0.04, 0.001**)	‐0.03 (‐0.08‐0.02, 0.243)
Covariates adjusted for:				
Sex	0.43 (0.20‐0.66, < 0.001***)	0.34 (0.08‐0.59, 0.009**)	0.34 (0.12‐0.57, 0.002**)	‐0.16 (‐0.39‐0.07, 0.173)
Age at baseline	‐0.01 (‐0.08‐0.06, 0.736)	‐0.02 (‐0.09‐0.06, 0.673)	‐0.04 (‐0.10‐0.03, 0.292)	< ‐0.01 (‐0.07‐0.07, 0.950)
Birthweight, kg	0.72 (0.46‐0.99, < 0.001***)	0.77 (0.48‐1.06, < 0.001***)	0.46 (0.21‐0.72, < 0.001***)	0.51 (0.24‐0.77, < 0.001***)
Iron status at baseline				
Fer <15 ug/L	0.39 (0.09‐0.69, 0.010*)	0.20 (‐0.14‐0.53, 0.249)	0.38 (0.09‐0.67, 0.011*)	0.31 (0.02‐0.61, 0.039*)
Fer >50 ug/L	‐0.07 (‐0.35‐0.20, 0.594)	‐0.24 (‐0.54‐0.07, 0.127)	0.04 (‐0.22‐0.31, 0.739)	‐0.12 (‐0.39‐0.15, 0.372)
Prelacteal feeding	< ‐0.01 (‐0.11‐0.11, 0.988)	‐0.06 (‐0.19‐0.06, 0.334)	0.05 (‐0.06‐0.15, 0.410)	‐0.01 (‐0.12‐0.11, 0.916)
Exclusive breastfeeding for more than 3 months	0.01 (‐0.15‐0.17, 0.905)	‐0.02 (‐0.20‐0.16, 0.856)	0.03 (‐0.13‐0.18, 0.732)	0.03 (‐0.13‐0.19, 0.687)
Started complementary feeding at less than 3 months	0.28 (‐0.11‐0.68, 0.161)	0.26 (‐0.19‐0.70, 0.258)	0.22 (‐0.17‐0.60, 0.271)	0.20 (‐0.20‐0.59, 0.328)
Still breastfeeding	‐0.05 (‐0.13‐0.02, 0.153)	‐0.05 (‐0.18‐0.07, 0.410)	‐0.11 (‐0.22‐0.01, 0.061)	‐0.06 (‐0.17‐0.04, 0.231)
Drank from a bottle with a nipple in the past 24hrs	0.07 (0.02‐0.11, 0.002**)	0.14 (0.06‐0.21, < 0.001***)	0.01 (‐0.05‐0.08, 0.708)	0.04 (‐0.02‐0.10, 0.157)
Minimum dietary diversity in previous 24hrs	0.02 (‐0.02‐0.05, 0.392)	0.04 (‐0.02‐0.11, 0.208)	‐0.01 (‐0.06‐0.05, 0.827)	‐0.01 (‐0.06‐0.05, 0.836)
Diarrhoea in past 2 weeks	‐0.09 (‐0.12 ‐ ‐0.05, < 0.001***)	‐0.02 (‐0.09‐0.04, 0.463)	‐0.10 (‐0.16 ‐ ‐0.05, < 0.001***)	‐0.10 (‐0.15 ‐ ‐0.04, < 0.001***)
Mother underweight at baseline (BMI<18.5)	‐0.28 (‐0.58‐0.03, 0.081)	‐0.02 (‐0.36‐0.33, 0.926)	‐0.32 (‐0.62 ‐ ‐0.03, 0.033*)	‐0.29 (‐0.60‐0.02, 0.064)
Caregiver attended				
Primary school	‐0.07 (‐0.47‐0.33, 0.723)	0.01 (‐0.43‐0.46, 0.949)	‐0.05 (‐0.43‐0.34, 0.812)	‐0.02 (‐0.42‐0.38, 0.915)
High school or higher	0.22 (‐0.17‐0.62, 0.269)	0.19 (‐0.25‐0.64, 0.390)	0.19 (‐0.20‐0.57, 0.335)	0.28 (‐0.12‐0.67, 0.171)
Poor card holder	‐0.22 (‐0.54‐0.09, 0.167)	‐0.34 (‐0.70‐0.01, 0.060)	‐0.02 (‐0.32‐0.29, 0.918)	‐0.20 (‐0.51‐0.11, 0.214)
Shared toilet	‐0.27 (‐0.59‐0.06, 0.104)	‐0.33 (‐0.69‐0.03, 0.072)	‐0.13 (‐0.44‐0.18, 0.415)	‐0.24 (‐0.56‐0.08, 0.147)

Mixed effects regression models were fit for each anthropometric measure, namely weight‐for‐age z‐score (WAZ), height‐for‐age z‐score (HAZ), weight‐for‐height z‐score (WHZ), and mid‐upper arm circumference (MUAC). Anthropometric measures were compared between the children in each pair of groups. Baseline values were accounted for in the model within each food group. Asterisks highlight significant *P*‐values:

* <0.05,

** < 0.01,

*** <0.001.

a "Month" refers to the control group and how long the control group has been on the program.

b This model includes parameters for the slope of the line in the control group with respect to time, and the change in the slope between the RUSF, CSB++ and MNP groups versus the control group.

c Missing data in the covariates resulted in a smaller n. In each cell, the coefficients, standard error and *P*‐value are reported.

## 3 RESULTS

**Figure 1:**
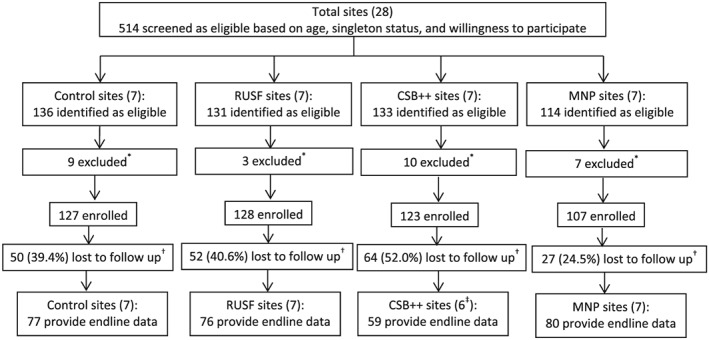
Trial profile‐site selection, recruitment, enrolment of children and trial completion. ^*^Reasons for exclusion: severe acute malnutrition, overnutrition, severe anaemia, food allergy. ^†^Reasons for loss to follow up: dropped out, moved away permanently, travelling temporarily, too busy to attend. ^‡^One site recruited only one child who dropped out, leaving 6 CSB++ sites and 27 sites in total

Of 514 children who were screened as eligible, 485 were recruited. Among 29 children excluded, one was excluded due to food intolerances, one due to severe anaemia, and 27 due to severe acute malnutrition (MUAC <11.5 and/or WHZ < ‐3) or overnutrition (WHZ > 3). Excluded children were referred for treatment as appropriate. See Figure. 1 for the site selection, recruitment and enrolment of children, and trial completion.

A total of 192 children (39.7%) did not attend endline. Loss to follow up ranged from 24.5% in the MNP group to 52.0% in the CSB++ group. There were differences in loss to follow up between the groups. The MNP group had the lowest loss, while the control, CSB++ and RUSF groups had significantly higher loss to follow up. Older children had slightly higher odds of being lost to follow up. Children whose caregivers had attended high school or higher had lower odds of being lost to follow up, as did children whose family were poor card holders. Details on loss to follow up can be found in Tables A3a and A3b.

### 3.1 Baseline characteristics

Table 1 describes the baseline characteristics of children and caregivers. For most characteristics, there were no significant differences between groups. However, the control and MNP groups had significantly more females. The CSB++ and MNP groups had significantly lower HAZ at baseline (however, the histogram showed sufficient overlap for the mixed effect model to account for this baseline difference). Infant feeding indicators were poor. Prevalence of prelacteal feeding was high, but lower than the Phnom Penh prevalence in the 2014 DHS (NIS et al., 2015). The prevalence of bottle feeding was very high while prevalence of continued breastfeeding at baseline was very low in comparison to the national prevalence (NIS et al., 2015). Rates of low birthweight (<2.5kg) were high in comparison to the national prevalence (NIS et al., 2015). Most children were iron replete, i.e. ferritin concentrations corrected for inflammation ≥ 15μg/L (Thurnham et al., 2010) at baseline. One‐third of children had experienced diarrhoea in the past 2 weeks. Unsafe disposal of children's faeces (left in the open or thrown in a drain or the garbage) was very high and was significantly different between groups.

### 3.2 Anthropometric outcomes

Table 2 shows the change in anthropometric measures from baseline to endline for children with baseline and endline measurements (n = 292). There were no statistically significant differences between the groups for any of the anthropometric changes. Mean height increased between 6.4‐6.7cm for all groups. Mean weight increased between 1.20 and 1.30kg for all groups. Mean WAZ, HAZ and WHZ decreased overall and for each group. Mean MUAC increased overall and for each group. The proportion of children underweight at endline was variable; it was unchanged in the RUSF group, increased in the CSB++ and MNP groups, and decreased in the control group. The proportions of children stunted and wasted increased in all groups. The proportion of children with low MUAC at endline compared with baseline decreased for the RUSF group, whereas for the other groups it increased or remained unchanged. There were no statistically significant differences between any of the changes in anthropometric measures. Figure A1 graphs the change in monthly mean anthropometric measures from baseline to endline.

A linear mixed effects model that took into account measures at each follow‐up was fitted for each anthropometric measure. The results of these models are shown in Table 3.

In unadjusted analysis, the control group had statistically significantly decreased WAZ and HAZ, and increased MUAC, but no statistically significant changes in WHZ. The RUSF group did not differ significantly from the control for WAZ or HAZ but had increased MUAC in comparison to the control. There were no statistically significant differences between the RUSF group and the CSB++ or MNP groups with respect to WAZ, HAZ, WHZ or MUAC. The CSB++ group did not differ significantly from the control for WAZ, HAZ or WHZ, but had a statistically significantly increased MUAC. The MNP group did not differ significantly from any group for WAZ, HAZ, WHZ or MUAC.

In the adjusted model, missing data in the covariates resulted in a smaller n (n = 235). The control group (Month) had statistically significantly decreased WAZ, HAZ, and WHZ, and no statistically significant change in MUAC. High consumers of RUSF did not differ significantly from the control for WAZ, HAZ or WHZ, but had statistically significantly increased MUAC. There were no significant differences between high consumers of RUSF and the CSB++ or MNP groups with respect to WAZ, HAZ, WHZ or MUAC. In comparison to the control, low consumers of RUSF had statistically significantly increased WAZ, WHZ and MUAC, but no statistically significant difference in HAZ. In comparison to the CSB++ group, low consumers of RUSF had statistically significantly increased HAZ, but no differences in other anthropometric measures. There were no statistically significant differences between low consumers of RUSF and the MNP group.

High consumers of CSB++ had statistically significantly increased WAZ, WHZ and MUAC in comparison to the control group, increased WHZ in comparison to the RUSF group, and increased WAZ and WHZ in comparison to the MNP group. Low consumers of CSB++ had statistically significantly increased WHZ and MUAC in comparison to the control, but decreased HAZ in comparison to all groups. High consumers of MNP had statistically significantly increased WAZ and MUAC in comparison to the control. Low consumers of MNP had no significant differences to the control for any anthropometric outcome but had decreased WAZ and WHZ in comparison to the RUSF and CSB++ groups.

Sex, birthweight, iron status, and diarrhoea significantly affected anthropometric status. Bottle feeding and maternal body mass index (BMI) were also significant. Age at baseline, iron repleteness at baseline, prelacteal feeding, cessation of exclusive breastfeeding before 3 months, age of commencing complementary feeding, continued breastfeeding, dietary diversity, caregiver's education, and living in a household that holds a poor card or shares a toilet did not have a significant effect on anthropometric outcomes.

## 4 DISCUSSION

In our trial, a locally produced, fish‐based RUSF slowed but did not prevent ponderal growth faltering in Cambodian children aged between 6 and 17 months. However, the impact was of limited clinical significance. The RUSF did not prevent linear growth faltering. Nor did CSB++ and MNP prevent growth faltering, or slow it to any clinically significant extent. This is consistent with studies elsewhere and in Cambodia, which have demonstrated the difficulty in preventing undernutrition in a representative population with moderately acutely malnourished (MAM) and non‐MAM children using specialised products.

### 4.1 Few trials in a representative, food secure population

Despite the consensus that prevention is essential, most specialised foods have been tested with MAM children (WHZ ‐3 to ‐2, and/or MUAC 11.5 to 12.5cm). Few prevention studies exist (Kennedy, Branca, Webb, Bhutta, & Brown, 2015), especially with non‐MAM children receiving a preventative specialised food in comparison to an unsupplemented control group. The children in our study ranged from MAM to overweight, i.e. WHZ 2 to 3 (WHO, 2006). In Cambodia, as in most countries, there is no treatment of children with MAM. Our sample of children had a similar prevalence of MAM as the general population of Cambodian children aged 6 to 17 months (NIS et al., 2015). It can therefore be considered representative of the general population that might be targeted for undernutrition prevention programming, in that this population includes some moderately acutely malnourished children and mostly children that range from WHZ >‐2 to <3 with MUAC > 12.5cm. This is not to say that the results can be generalised. One systematic review used the concept of food security and by their definition (Lassi, Das, Zahid, Imdad, & Bhutta, 2013), our population could be considered to be in a food secure, non‐emergency context.

### 4.2 Do specialised products prevent undernutrition?

In our trial, specialised products had limited effect on reducing growth faltering, as seen in Tables 2 and 3. To some extent, all the specialised products in our trial, especially the RUSF and CSB++ protected against ponderal growth faltering, but none protected against linear growth faltering. The RUSF afforded more protection than MNP, but not more than CSB++. In comparable trials, the impact of supplementary feeding on undernutrition has often been similarly slight, mixed, or nonsignificant. In those trials, WAZ, WHZ and MUAC usually increased for at least one of the intervention groups, whereas HAZ was less likely to improve and sometimes declined (Dewey & Adu‐Afarwuah, 2008; Iannotti et al., 2014; Lin et al., 2008; Lutter et al., 2008; Ruel et al., 2008; Sguassero, de Onis, Bonotti, & Carroli, 2012; Skau et al., 2015; Thakwalakwa et al., 2012; Tomedi et al., 2012). One study, like ours, found HAZ decreased more for the CSB++ group than for the control (Mangani et al., 2015). However, it is worth noting that HAZ was already significantly lower in the CSB++ group at baseline.

Therefore, most interventions providing supplements or specialised foods did not prevent stunting, and some did not even prevent wasting. Hence, that the interventions in our study did not prevent growth faltering, and only had a small impact on anthropometry in comparison to the control was not unprecedented. A forthcoming Cochrane Review (see the protocol by Das, Salam, Weise Prinzo, Sadiq Sheikh, & Bhutta, 2017) will assess the effects of preventive lipid‐based nutrient supplements given with complementary foods to infants and young children. This will contribute greatly to the understanding of the effects of specially formulated supplementary foods.

### 4.3 Diarrhoea

One possible explanation for the continued growth faltering observed in our study is that the nutrients from both the standard diet and the interventions provided may not have been well absorbed. Children who had had diarrhoea in the past 2 weekshad decreased WAZ, WHZ and MUAC. The prevalence of diarrhoea in our study population (32% overall) was much higher than the prevalence of diarrhoea in children under 5 years in Phnom Penh (17%) or nationally to children aged 6 to 11 months or twelve to 23 months (20% and 19%, respectively). However, it was similar to the prevalence of diarrhoea (40% of children under 5 years) in a comparable survey amongst urban poor in Phnom Penh (UNICEF/PIN , 2014). Thus, high rates of diarrhoea may have contributed to continued growth faltering. Unsafe stool disposal was common, and may contribute to high rates of diarrhoea.

### 4.4 Sex

Another explanation may be related to sex. In our trial, female children had increased WAZ, HAZ and WHZ compared with male children. The control and MNP groups had significantly more females. Since gender has been found to have a differential impact on MUAC and WHZ, particularly in the presence of stunting (Fiorentino et al., 2016; Wieringa et al., 2018), this may explain why a greater difference was not seen between the outcomes for the RUSF and CSB++ groups compared with the control and MNP groups.

### 4.5 Potential displacement of breastmilk and food

Another possible explanation for the lack of effect on prevention of growth faltering may be that RUSF and CSB++ may have displaced children's normal intake of food and breastmilk rather than actually supplementing the existing diet (Dewey & Adu‐Afarwuah, 2008; Mangani et al., 2015). The quantities of RUSF and CSB++ given in our study (between 40‐110g/day) were relatively large and could conceivably have displaced breastmilk and other family foods (Dewey & Arimond, 2012). However, analysis thus far on the displacement of breastmilk and family food does not reveal any difference between dietary intakes across the groups (see Table A4).

### 4.6 Other explanations for growth faltering

In our trial, children with higher birthweight had significantly greater increase in WAZ, HAZ, WHZ and MUAC from baseline to endline. Children of underweight mothers (BMI < 18.5 at baseline) had decreased WHZ. This highlights the multifactorial causes of child undernutrition. Additional factors, including birthweight, maternal BMI, iron status, and diarrhoea which contribute to poor anthropometric outcomes, must be taken into consideration, along with interventions to address them, such as maternal supplementation and adequate antenatal care, delayed cord clamping, and diarrhoeal prevention and treatment (Bhutta et al., 2013).

### 4.7 Non‐milk animal source foods

Daily consumption of animal‐source foods is recommended for providing the protein, energy, and micronutrients needed for healthy micronutrient status, linear and ponderal growth (Manary, 2012; Michaelsen, Grummer‐Strawn, & Begin, 2017; Neumann et al., 2013; PAHO/WHO, 2002). Most RUFs use milk or whey; non‐milk supplementary foods using meat, fish or eggs have rarely been compared with milk‐based products (Anderson, Bediako‐Amoa, & Steiner‐Asiedu, 2014; Bogard et al., 2015; Gera et al., 2017; Kuusipalo et al., 2006; Pachón, Domínguez, Creed‐Kanashiro, & Stoltzfus, 2007; Skau et al., 2014). However, the evidence on whether milk or other animal source foods are more effective in preventing undernutrition is mixed. Two efficacy studies have involved fish‐based supplementary foods. In Malawi, a study comparing a corn porridge fortified with fish powder to a peanut/soy spread found that children had similar linear and ponderal growth (Lin et al., 2008). In Cambodia, Winfood, based on rice and fish, was compared with CSB++ (containing milk) and CSB+ (containing no milk). Both Winfood and CSB++ promoted linear growth better than CSB+ (Skau et al., 2015). One study that compared milk and meat found meat had a greater impact (Grillenberger et al., 2003). In our trial, both the fish‐based RUSF and the milk‐based CSB++ provided some protection against ponderal growth faltering, demonstrating that fish has the potential to replace milk in specialised foods.

### 4.8 Micronutrients and macronutrients

Our study is consistent with trials that found that in the absence of adequate macronutrients, micronutrients alone do not contribute to growth (Adu‐Afarwuah et al., 2007; Dewey & Adu‐Afarwuah, 2008; Dewey, Yang, & Boy, 2009; Imdad, Sadiq, & Bhutta, 2011; Jack et al., 2012; Rivera & Habicht, 2002; Zlotkin, 2009). Children in the high consuming MNP group had increased WAZ and MUAC compared with the control in a similar magnitude to the RUSF and CSB++ groups. Low consumers of MNP had no significant differences to the control for any anthropometric outcome, and had poorer outcomes for WAZ, HAZ and WHZ than children in the RUSF and CSB++ groups. Since MNP is added to food, these results may be interpreted as children who are high consumers of MNP actually eating more food, thus receiving the necessary macronutrients along with the MNP micronutrients.

### 4.9 High and low consumption

In our trial, low rather than high consumers of RUSF experienced a protective effect against faltering of WAZ, WHZ and MUAC. This suggests that the RUSF, even in small quantities, actually supplements the existing diet as intended. Other researchers who have worked on small quantity LNSs (20‐50g/day) have found that in small quantities, LNSs may improve growth (Dewey et al., 2017; Hess et al., 2015). They may also improve appetite (Arimond et al., 2015; Lesorogol, Jean‐Louis, Green, & Iannotti, 2015), something which caregivers in our acceptability trial remarked upon (Borg et al., 2018). This finding warrants a trial of the RUSF in small quantities.

That most plausible interpretation of the increased weight‐related anthropometric measures (WAZ, WHZ and MUAC) among high consumers of CSB++ and MNP in comparison to the control group is that high consumers are eating more food generally. Hence it would be expected that their growth would falter less than the control group.

### 4.10 Strengths and limitations

This study had two main strengths. First, this is one of few undernutrition prevention trials that has compared a novel specially formulated supplementary food to an unsupplemented control group, as well as to CSB++ and MNP which are widely used specialised products. Use of an unsupplemented control enables the assessment of the clinical and programmatic significance of the results. It informs programming, by making it possible to compare the provision of specialised products to no intervention (Gera et al., 2017). Second, our study generated much needed evidence in a geographic and social context other than Africa (Gera et al., 2017; Kennedy et al., 2015; Lazzerini et al., 2013).

There are four main limitations of this trial. First, the high and differential loss to follow up may have introduced bias. Second, self‐reporting on compliance favours over‐reporting of consumption, which may lead to underestimation of effectiveness. Third, our findings may not be generalisable to non‐urban Cambodian populations. Since rural areas of Cambodia experience higher levels of undernutrition and poorer infant and young child feeding practices (NIS et al., 2015), it would be difficult to predict if the interventions would appear more or less effective. Finally, subgroup analysis of the effect of the specialised products specifically on MAM children was not undertaken due to low sample size.

## 5 CONCLUSION

Our trial contributes to the limited literature on the supplementation of a population sample of children in a food secure, non‐emergency setting. This makes it useful for programming, which has had to rely on findings from studies that focus specifically on MAM children or food insecure settings. In this trial, the most important finding is that the locally produced, fish‐based RUSF, consumed in small quantities, was superior to a standard diet. In small quantities, the RUSF protected against the wasting and underweight seen in the control group, with improved outcomes for WAZ, WHZ and MUAC. However, the magnitude of improvements was of limited clinical significance

There were few significant differences between the RUSF and the CSB++ or MNP groups. None of the specialised products protected against stunting. The RUSF was not superior to CSB++. Both the RUSF and CSB++ groups performed better than low consumers of MNP, which confirms earlier findings that micronutrients in the absence of macronutrients do not improve growth. However, once again, the magnitude of improvements was of limited clinical significance.

Further research is warranted to explore the potential role, if any, of supplements and specially formulated supplementary foods in preventing undernutrition in a representative population of Cambodian children. With respect to the RUSF, future trials with MAM children, and with small quantities of the RUSF may be warranted. All future studies should include a control with a standard, unsupplemented diet. Programming for the prevention of childhood undernutrition in Cambodia will need to consider other approaches and address additional important factors. These findings should assist programmers in selecting nutrition interventions.

## CONFLICTS OF INTEREST

The authors declare that they have no conflicts of interest.

## ETHICAL STATEMENT

Ethics approval was received from the University of Queensland Medical Research Ethics Committee (2014001070) and from Cambodia's National Ethics Committee for Health Research (402 NECHR).

## CONTRIBUTIONS

BB developed the research protocol, trial design, and questionnaires, and refined these with FTW, SM, MG, DS, CC, JB, AL and NR. AL and FTW secured funding. BB managed data collection with DS. BB conducted the statistical analysis with support from MG. BB wrote the manuscript and all authors subsequently commented on the manuscript and approved the final version.

## CLINICAL TRIAL NUMBER

http://ClinicalTrials.gov, Identifier: LNS‐CAMB‐INFANTS‐EFF; NCT02257762.

## DATA SHARING

The datasets generated and/or analysed during the current study will be made available from the corresponding author after the publication of all major outputs, upon reasonable request.

## Supporting information


**Supporting Information**
