# Supplementary material for: Effectiveness of a locally produced ready‐to‐use supplementary food in preventing growth faltering for children under 2 years in Cambodia: a cluster randomised controlled trial
Source: Matern Child Nutr. 2019 Dec 29;16(1):e12896. doi: 10.1111/mcn.12896 (PMC7038903; doi:10.1111/mcn.12896)
Supplement: Supplementary file 1 — Supporting Information [file MCN-16-e12896-s001.pdf]

MCN12896 Appendices combined

Appendix Table 1: Ingredients of RUSF snack (paste and wafer)

**Ingredients**	**g/100g**
Small indigenous fish	5.9
Mung beans	9.6
Rice	4.2
Soy beans	12.2
Icing sugar	10.3
Maltrodextrin	9.3
Canola oil	3.7
Palm vegetable shortening	14.0
Desiccated coconut	1.5
Rice bran	2.2
Vitamin and mineral mix	0.9
Rice flour	9.0
Duck eggs	2.5
Refined sugar	7.2
Coconut	7.2
Salt	0.0
Flavour (vanilla or sesame seeds)	0.1
Oil for cooking	0.4

Appendix Table 1: Ingredients of RUSF snack (paste and wafer)

Appendix Table 2: Characteristics of the RUSF and comparators

CHARACTERISTIC	RUSF	CSB++	MNP
**Daily serving size**	40-110g*	100g dry CSB++	1 sachet (1g)
**Animal-source food**	Fish	Milk	-
**Energy (kcal/100g)**	484	410	-
**Protein (g/100g)**	13	16	-
**Carbohydrates (g/100g)**	52	67	-
**Lipids (g/100g)**	24	9	-
**Fibre (g/100g)**	1.6	3	-
**Vitamin A**	1,080 µg	540 µg	400 µg
**Vitamin D**	60 µg	4.6 µg	5 µg
**Vitamin B1 (thiamine)**	0.59 mg	0.47 mg	0.5 mg
**Vitamin B2 (riboflavin)**	0.89 mg	0.84mg	0.5 mg
**Vitamin B6**	0.84 mg	2.1 mg	0.5 mg
**Phosphorus**	474 mg	530 mg	-
**Calcium**	366 mg	260 mg	-
**Pantothenic acid**	1.75 mg	7.3 mg	-
**Copper**	1.6 mg	-	0.56 mg
**Vitamin E**	10.9 mg	9.8 mg	5 mg
**Folic acid**	230 µg	115 µg	150 µg
**Iron**	8 mg	8.9 mg	10 mg
**Magnesium**	137 mg		-
**Vitamin B3 (niacin)**	9.63 mg	7.2 mg	6 mg
**Vitamin C**	53.4 mg	100 mg	30 mg
**Zinc**	8.4 mg	7.5 mg	4.1 mg
**Potassium**	806 mg	990 mg	-
**Vitamin B12**	10 µg	2.3 µg	0.9 µg
**Biotin**	0.37 mg	-	-
**Selenium**	90 µg	-	17 µg
**Iodine**	-	60 mg	90 µg
**Vitamin K**	3 µg	115 µg	-
**Taste**	Fishy	Creamy, sweet, smooth (Skau, Sok & Wieringa, 2012)	Should not have a taste (Salam, Macphail, Das, & Bhutta, 2013)
**Preparation**	No	10 mins cooking	No
**Acceptability in Cambodia**	Yes (Borg et al., 2019)	Acceptable in trial (Skau, Sok & Wieringa, 2012), but not in practice (WFP, 2014)	Yes (Jack et al., 2012)
**Effectiveness in reducing malnutrition**	To be tested	Not inferior to peanut-based RUSFs, which are the most effective in promoting linear growth and weight gain (LaGrone et al., 2012; Manary & Chang, 2012.)	Improves micronutrient status but not linear growth or weight gain (de Pee & Bloem, 2009; Dewey & Adu-Afarwuah, 2008; Jack et al., 2012)
**Intra-household sharing**	Unknown	Yes (LaGrone et al., 2012)	None noted (Jack et al., 2012)

**Packaging**	Unknown	Packaging may encourage sharing (de Pee & Bloem, 2009; Nackers et al., 2010)	Looks like "medicine" thus may discourage sharing (de Pee & Bloem, 2009; Nackers et al., 2010)
**Local production capacity**	Unknown	None (de Pee & Bloem, 2009)	None
**Cost**	To be determined. Goal is <US$0.10/day	Less expensive than peanut-based RUSFs if produced locally (Manary & Chang, 2012.), but also have to consider logistics, time to treat, relapse (Nackers et al., 2010)	Very cheap to produce at US$0.025/daily dose (Zlotkin, 2009), but also have to consider logistics

Appendix Table 2: Characteristics of the RUSF and comparators
** RUSF daily serving size depends on the child's age, i.e. 6-8m – 4 pieces, 40g; 9-11m – 6 pieces, 60g; 12-17m – 11 pieces, 110g.*

Comparators
The RUSF was compared with:

1. CSB++: CSB++ was chosen as a comparator because it is the standard supplementary food that WFP provides to children aged six months to two years to prevent undernutrition.
2. MNP: Sprinkles micronutrient powders were chosen since they are a commonly provided supplement in developing countries, such as Cambodia, with low dietary diversity, and complementary foods with low nutrient density (HF-TAG, 2011)
3. Control: A standard, unsupplemented diet, typically *borbor* and family foods was chosen as a control. *Borbor* is the traditional food for weanlings (children transitioning from exclusively milk diets to diets that include complementary foods) and is often the only food given until about nine months.

The active comparators complied with WFP and UNICEF standards for supplementary foods, and had been used and tested in Cambodia and elsewhere (Jack et al., 2012; LaGrone et al., 2012; Manary & Chang, 2012.). They have been found to be safe and to have no unintended side-effects. Table 2 contrasts the characteristics of the RUSF and comparators.

Plumpy'Nut™ was a potential comparator that was not used because it was less acceptable (Boudier, 2009). Moreover, including peanuts in a locally produced Cambodian RUSF was not considered advisable due to the high risk of aflatoxin contamination in South-East Asia (Binder et al., 2007; Shank, Wogan, & Gibson, 1972; Tran-Dinh, Kennedy, Bui, & Carter, 2009).

Binder, E. M., Tan, L. M., Chin, L. J., Handl, J., & Richard, J. (2007). Worldwide occurrence of mycotoxins in commodities, feeds and feed ingredients. Animal Feed Science and Technology, 137(3), 265-282.

Borg, B., Mihrshahi, S., Griffin, M., Sok, D., Chhoun, C., Laillou, A., & Wieringa, F. T. (2019). Acceptability of locally-produced Ready-to-Use Supplementary Food (RUSF) for children under two years in Cambodia: A cluster randomised trial. Maternal & Child Nutrition, e12780-e12780. doi:10.1111/mcn.12780

Boudier, F. (2009). Socio-anthropological Investigation Related to the Acceptability of Plumpy'nut® in Cambodia.

de Pee, S., & Bloem, M. W. (2009). Current and potential role of specially formulated foods and food supplements for preventing malnutrition among 6-to 23-month-old children and for treating moderate malnutrition among 6-to 59-month-old children. Food Nutr Bull, 30(3), S434-S463.

Dewey, K. G., & Adu-Afarwuah, S. (2008). Systematic review of the efficacy and effectiveness of complementary feeding interventions in developing countries. Maternal & Child Nutrition, 4 Suppl 1(s1), 24-85. doi:10.1111/j.1740-8709.2007.00124.x

HF-TAG. (2011). Programmatic Guidance Brief on Use of Micronutrient Powders (MNP) for Home Fortification.

Jack, S. J., Ou, K., Chea, M., Chhin, L., Devenish, R., Dunbar, Eang, Cl, Hou, K., Ly, S., Khin, M., Prak, S., Reach, R., Talukder, A., Tokmoh, L., Leon de la Barra, S., Hill, P. C., Herbison, P. M., Gibson, R. S. (2012). Effect of Micronutrient Sprinkles on Reducing Anemia. Archives of Pediatrics & Adolescent Medicine, 166(9), 842-850. doi:10.1001/archpediatrics.2012.1003

LaGrone, L. N., Trehan, I., Meuli, G. J., Wang, R. J., Thakwalakwa, C., Maleta, K., & Manary, M. J. (2012). A novel fortified blended flour, corn-soy blend "plus-plus," is not inferior to lipid-based ready-to-use supplementary foods for the treatment of moderate acute malnutrition in Malawian children. American Journal of Clinical Nutrition, 95(1), 212-219. Retrieved from doi:10.3945/ajcn.111.022525

Manary, M., & Chang, C. Y. (2012.). Comparing Milk Fortified Corn-Soy Blend (CSB++), Soy Ready-to-Use Supplementary Food (RUSF), and Soy/Whey RUSF (Supplementary Plumpy®) in the Treatment of Moderate Acute Malnutrition.

Nackers, F., Broillet, F., Oumarou, D., Djibo, A., Gaboulaud, V., Guerin, P. J., Captier, V. (2010). Effectiveness of ready-to-use therapeutic food compared to a corn/soy-blend-based pre-mix for the treatment of childhood moderate acute malnutrition in Niger. Journal of tropical pediatrics, 56(6), 407-413. doi:10.1093/tropej/fmq019

Salam, R. A., Macphail, C., Das, J. K., & Bhutta, Z. A. (2013). Effectiveness of Micronutrient Powders (MNP) in women and children. BMC public health, 13 Suppl 3(Suppl 3), S22. doi:10.1186/1471-2458-13-S3-S22

Shank, R. C., Wogan, G. N., & Gibson, J. B. (1972). Dietary aflatoxins and human liver cancer. I. Toxigenic moulds in foods and foodstuffs of tropical south-east asia. Food and Cosmetics Toxicology, 10(1), 51-60. doi:10.1016/S0015-6264(72)80046-4

Skau, J. N., M.; Sok, D.; Wieringa, F. (2012). Acceptability Study Of Fortified Blended Food Products Among Children 12-36 Months Old Children And Caregivers In Cambodia (unpublished).

Tran-Dinh, N., Kennedy, I., Bui, T., & Carter, D. (2009). Survey of Vietnamese peanuts, corn and soil for the presence of Aspergillus flavus and Aspergillus parasiticus. Mycopathologia, 168(5), 257-268. doi:10.1007/s11046-009-9221-9

WFP. (2014). [Personal communication].

Zlotkin, S. (2009). Overview of Efficacy, Effectiveness and Safety of MNPs. Retrieved from http://www.unicef.org/nutritioncluster/files/Revised_UNICEF_CombinedOverview_of_Efficacy_Effectiveness_and_Safety_of_MNPs.pdf. (Accessed 11 December 2015).

Appendix Tables 3a and 3b: Loss to follow up

	Total (N=485)	Control (n=127, 26%)	RUSF (n=128, 26%)	CSB++ (n=123, 25%)	MNP (n=106, 22%)	P-value
**Loss to follow up, n (%)**	192 (39.7%)	50 (38.4%)	52 (40.6%)	64 (52.0%)	26 (24.5%)	< 0.001***

Appendix Table 3a: Loss to follow up across the arms from baseline to endline.
*P-value was computed using Pearson chi squared. Asterisks highlight significant p-values: * <0.05, ** < 0.01, ***<0.001.*

**Loss to follow up**	**Odds ratio**	**95% CI**	**P value**
MNP vs control	2.37	1.03, 5.44	0.042*
MNP vs RUSF	3.89	1.71, 8.88	0.001**
MNP vs CSB++	4.84	2.08, 11.29	<0.000***
Control vs RUSF	1.65	0.76, 3.55	0.204
Control vs CSB++	2.05	0.92, 4.57	0.081
RUSF vs CSB++	1.24	0.57, 2.72	0.585
**Adjusted for:**			
Month of study	0.59	0.54, 0.63	<0.000***
Sex	0.90	0.70, 1.17	0.443
Age at baseline	1.12	1.04, 1.20	0.002**
Caregiver attended			
- primary school	1.28	0.84, 1.96	0.252
- high school or higher	0.64	0.41, 0.99	0.047*
Poor card holder	0.51	0.34, 0.77	0.001**
Diarrhoea	0.87	0.66, 1.14	0.305
Random effects	0.42	0.19, 0.94	

Appendix Table 3b: Loss to follow up from baseline to endline.
*Odds ratios, standard errors P-values, and 95% Confidence Intervals were computed using mixed effects regression models. Asterisks highlight significant p-values: * <0.05, ** < 0.01, ***<0.001.*

All subjects (N=485) attended baseline. Thereafter, subjects attended 60-75% of data collection sessions. Loss to follow up refers to subjects who failed to attend the endline data collection (n = 192), regardless of how many other data collection points they attended. There were significant differences in loss to follow up between the groups. The MNP group had significantly lower odds of being lost to follow up. Comparisons of the odds of dropping out between the other groups were not statistically significant.

In comparison to the MNP group, subjects in the control group had more than twice the odds of dropping out (OR = 2.37; 95% CI = 1.03, 5.44; p = 0.042), while RUSF subjects had almost four times the odds (OR = 3.89; 95% CI = 1.71, 8.88; p = 0.001), and CSB++ subjects had almost five times the odds of dropping out (OR = 4.84; 95% CI = 2.08, 11.29; p <0.001). In comparison to the control group, subjects in the RUSF and CSB++ groups had about twice the odds of dropping out (OR = 1.65; 95% CI = 0.76, 3.55; p = 0.204; and OR = 2.05; 95% CI = 0.92, 4.57; p = 0.081 respectively) although these were not statistically significant. In comparison to the RUSF group, subjects in the CSB++ groups had slightly greater odds of dropping out (OR = 1.24; 95% CI = 0.57, 2.72; p = 0.585) although this was not statistically significant.

For every additional month that a subject stayed in the study, their odds of dropping out decreased by approximately half (OR = 0.58; 95% CI = 0.54, 0.62; p<0.001). Subjects who were older at baseline had slightly higher odds of dropping out (OR = 1.12; 95% CI = 1.04, 1.01; p=0.002). Subjects whose caregiver had had attended high school or higher had lower odds of dropping out (OR = 0.64; 95% CI = 0.41, 0.99; p=0.047). Subjects whose family were poor card holders had half the odds of dropping out (OR = 0.51; 95% CI = 0.34, 0.77; p=0.001). Sex, primary school education and having diarrhoea in the past two weeks did not make a statistically significant difference.

Appendix Figure 1: Mean anthropometric measures and confidence intervals monthly from baseline to endline for children with baseline and endline measurements.

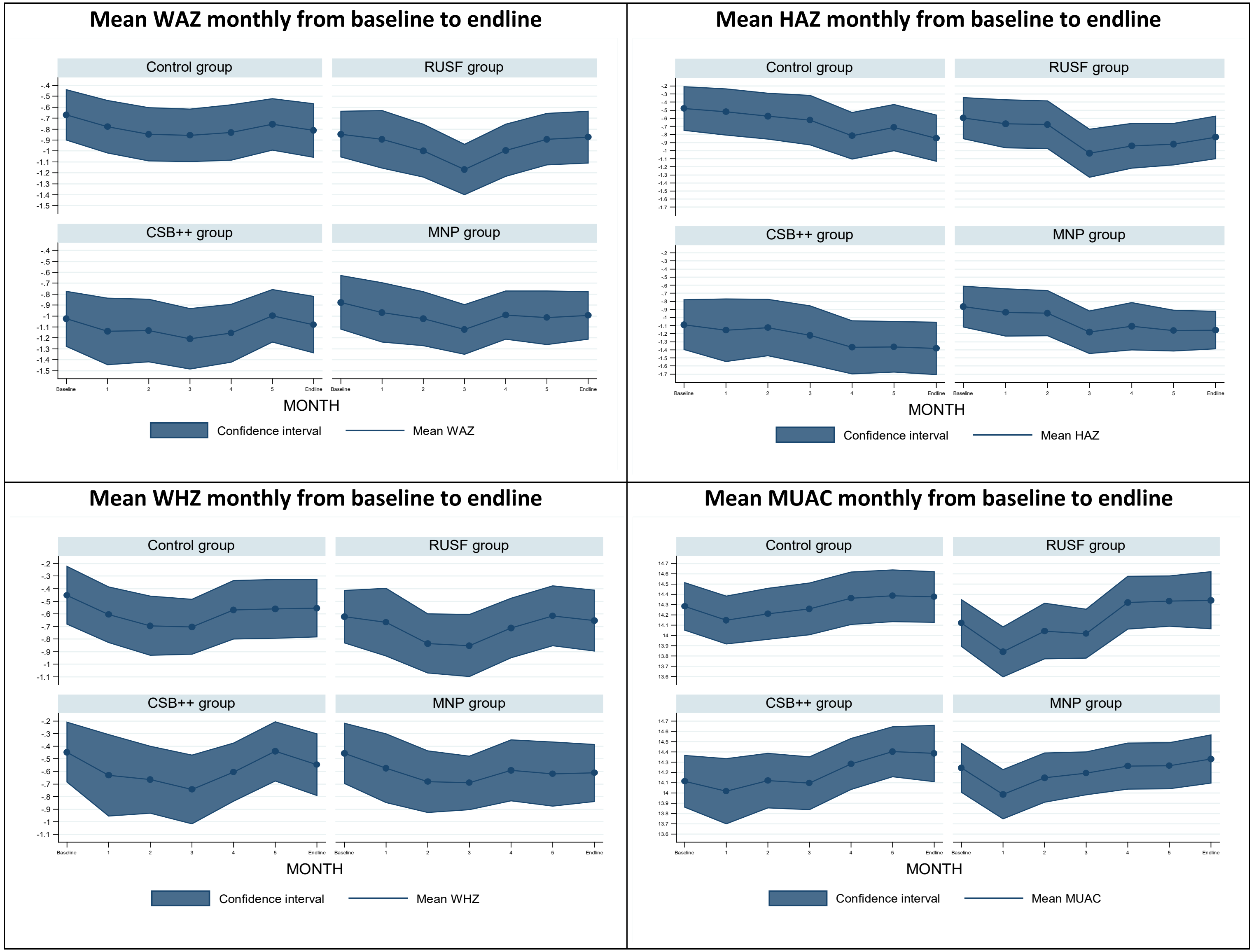

Appendix Figure 1: Mean anthropometric measures and confidence intervals monthly from baseline to endline for children with baseline and endline measurements

Appendix Figure 1 depicts the change in monthly mean anthropometric measures from baseline to endline for the 292 children with baseline and endline measurements. The graphs show that WAZ, HAZ and WHZ decline and MUAC increases. The wide and overlapping confidence intervals of the graphs indicate that there may be no significant difference between the groups.

Appendix Table 4: Change in dietary intake from baseline to endline

Change in dietary intake from baseline to endline	Total (N=292)	Control (n=77, 26%)	RUSF (n=76, 26%)	CSB++ (n=59, 20%)	MNP (n=80, 27%)	P-value
**Breastfeeding**						
At baseline, n (%)	187 (64.5%)	51 (66.2%)	47 (66.1%)	39 (62.7%)	50 (63.3%)	0.955
At endline, n (%)	144 (49.7%)	41 (54.0%)	33 (43.4%)	30 (50.9%)	40 (50.6%)	0.614
**Minimum dietary diversity in past 24hrs**						
At baseline, n (%)	81 (27.7%)	19 (24.7%)	24 (31.6%)	19 (32.2%)	19 (23.8%)	0.544
At endline, n (%)	256 (87.7%)	63 (81.8%)	68 (89.5%)	55 (93.2%)	70 (87.5%)	0.226
**Meal frequency in past 24hrs at baseline, n (%)**						
1-2 times	75 (26.8%)	24 (32.9%)	16 (21.9%)	9 (15.3%)	26 (34.7%)	0.004
3-4 times	200 (71.4%)	49 (67.1%)	57 (78.1%)	46 (78.0%)	48 (64.0%)	0.004
> 5 times	5 (1.8%)	0 (0.0%)	0 (0.0%)	4 (6.8%)	1 (1.3%)	0.004
**Meal frequency in past 24hrs at endline, n (%)**						
1-2 times	22 (7.5%)	7 (9.1%)	8 (10.5%)	2 (3.4%)	5 (6.3%)	0.812
3-4 times	262 (89.7%)	67 (87.0%)	66 (86.8%)	56 (94.9%)	73 (91.3%)	0.812
> 5 times	6 (2.1%)	2 (2.6%)	2 (2.6%)	1 (1.7%)	1 (1.3%)	0.812
**Amount eaten at each meal at baseline, n (%)**						
<2 tablespoonfuls each time	73 (26.0%)	19 (26.0%)	19 (25.7%)	15 (25.4%)	20 (26.7%)	0.866
2-3 tablespoonfuls each time	79 (28.1%)	25 (34.3%)	18 (24.3%)	13 (22.0%)	23 (30.7%)	0.866
< 1/2 bowl each time	78 (27.8%)	18 (24.7%)	22 (29.7%)	18 (30.5%)	20 (26.7%)	0.866
about 1 bowl each time	45 (16.0%)	9 (12.3%)	13 (17.6%)	13 (22.0%)	10 (13.3%)	0.866
>1 bowl each time	6 (2.1%)	2 (2.7%)	2 (2.7%)	0 (0.0%)	2 (2.7%)	0.866
**Amount eaten at each meal at endline, n (%)**						
<2 tablespoonfuls each time	19 (6.5%)	5 (6.5%)	4 (5.3%)	4 (6.8%)	6 (7.5%)	0.584
2-3 tablespoonfuls each time	81 (27.7%)	22 (28.6%)	19 (25.0%)	13 (22.0%)	27 (33.8%)	0.584
< 1/2 bowl each time	35 (12.0%)	12 (15.6%)	8 (10.5%)	8 (13.6%)	7 (8.8%)	0.584
about 1 bowl each time	150 (51.4%)	36 (46.8%)	41 (54.0%)	33 (55.9%)	40 (50.0%)	0.584
>1 bowl each time	6 (2.1%)	1 (1.3%)	4 5.3%)	1 (1.7%)	0 (0.0%)	0.584
**Consumed sweet or salty snacks (eg chips, cakes, candies) in the past 24hrs**						
At baseline, n (%)	86 (29.5%)	12 (15.6%)	26 (34.2%)	17 (28.8%)	31 (38.8%)	0.010
At endline, n (%)	249 (85.3%)	62 (80.5%)	63 (82.9%)	52 (88.1%)	72 (90.0%)	0.316

Appendix Table 4: Change in dietary intake from baseline to endline
P-values were computed by comparison of different food types using chi-squared.

There were no statistically significant differences between the groups in breastfeeding, dietary diversity, or amount eaten at each meal at baseline or endline. This suggests that the specialised foods did not displace breastmilk or food.

There was a statistically significant difference in meal frequency at baseline. More children in the control and MNP groups ate infrequently (1-2 times/day). At endline, there was no difference between groups. A possible explanation for the difference is that caregivers in the RUSF and CSB++ groups did not consider the specialised food a meal, and did not "count" them in answering the question at endline. If this were the case, it would mean that RUSF and CSB++ replaced meals. However, further analysis would be necessary to confirm that interpretation.

There was a statistically significant difference in consumption of snacks at baseline. Less children in the control group and more children in the MNP group ate snacks. At endline, there was no difference between groups. The question did not ask specifically about commercial snacks, so it cannot be confirmed whether parents in the RUSF or CSB++ groups considered the specialised foods as snacks.
